# Reduced live birth rates in frozen versus fresh single cleavage stage embryo transfer cycles: A cross -sectional study

**DOI:** 10.18502/ijrm.v13i7.7366

**Published:** 2020-07-22

**Authors:** Wan Tinn Teh, Alex Polyakov, Claire Garrett, David Edgar, John Mcbain, Peter Adrian Walton Rogers

**Affiliations:** ^1^Department of Obstetrics and Gynaecology, University of Melbourne, The Royal Women’s Hospital, Parkville, Victoria, Australia.; ^2^Reproductive Services, The Royal Women’s Hospital, Parkville, Victoria, Australia.; ^3^Melbourne IVF, East Melbourne, Victoria, Australia.

**Keywords:** Fresh, Frozen embryo transfer, Live birth, Embryo, Transfer.

## Abstract

**Background:**

Studies have suggested that embryo-endometrial developmental asynchrony caused by slow-growing embryos can be corrected by freezing the embryo and transferring it back in a subsequent cycle. Therefore, we hypothesized that live birth rates (LBR) would be higher in frozen embryo transfer (FET) compared with fresh embryo transfers.

**Objective:**

To compare LBR between fresh and FET cycles.

**Materials and Methods:**

A cross-sectional analysis of 10,744 single autologous embryo transfer cycles that used a single cleavage stage embryo was performed. Multivariate analysis was performed to compare LBR between FET and fresh cycles, after correcting for various confounding factors. Sub-analysis was also performed in cycles using slow embryos.

**Results:**

Both LBR (19.13% vs 14.13%) and clinical pregnancy (22.48% vs 16.25%) rates (CPR) were higher in the fresh cycle group (p < 0.00). Multivariate analysis for confounding factors also confirmed that women receiving a frozen-thawed embryo had a significantly lower LBR rate compared to those receiving a fresh embryo (OR 0.76, 95% CI 0.68-0.86, p < 0.00). In the sub-analysis of 1,154 cycles using slow embryos, there was no statistical difference in LBR (6.40% vs 6.26%, p = 0.92) or CPR (8.10% vs 7.22%, p = 0.58) between the two groups.

**Conclusion:**

This study shows a lower LBR in FET cycles when compared to fresh cycles. Our results suggest that any potential gains in LBR due to improved embryo-endometrial synchrony following FET are lost, presumably due to freeze-thaw process-related embryo damage.

## 1. Introduction

Prerequisites for successful implantation are an embryo with implantation competency, a receptive endometrium, and synchronous development between the embryo and the endometrium. (1-4). The relative contributions of reduced embryo viability, a non-receptive endometrium, or embryo-endometrial asynchronous to implantation failure following IVF are unknown. There have been statistical analyses that suggest both embryo viability and uterine receptivity play significant roles (5, 6). Another approach to investigating these issues is to compare pregnancy outcomes following fresh versus frozen embryo transfers (FETs).

It is widely believed that controlled ovarian hyperstimulation (COH) used in IVF cycles has detrimental effects on the endometrium, as well as potentially disrupting normal synchronous development between the endometrium and the embryo (7-17). It has been shown that pregnancy is more likely when there are fewer endometrial histological alterations after COH (18). No pregnancy was reported if development of the endometrium was greater than 3 days more advanced than the embryos (13, 14, 19).

Evidence that synchronous development between the embryo and the endometrium is important for successful implantation comes from work comparing normal-growing and slow-growing embryos on implantation rates and pregnancy rates (PR) between fresh autologous and FET cycles. As expected, the clinical PR was higher for normal-growing than the slow-growing embryos in fresh cycles (51% vs. 33.3%). However, if the slower blastocyst growth rates are compensated for by transferring on developmental age (day 5) rather than chronological age (day 6), then there was no significant difference in The PR between the normal- and slow-growing cryopreserved blastocysts following FET cycles (63.6% vs. 58.9%). Slow embryos were also associated with a significantly greater PR in FET cycles than in fresh autologous cycles (58.9% vs. 33.3%). (20) This study supports the hypothesis that embryo-endometrial developmental asynchrony caused by slow-growing embryos can be corrected by freezing the embryo and transferring it back a day earlier in a subsequent cycle.

Further support for FET giving improved results to fresh transfers comes from a recent randomized multicenter trial involving women with polycystic ovarian syndrome (PCOS) (21). This study demonstrated a higher live birth rate (LBR; 49.3% vs 42.0%) with FET than fresh embryo transfer cycles.

The primary aim of this retrospective study of 10,744 single embryo transfers from the Melbourne IVF (MIVF) database was to compare pregnancy outcomes between fresh versus frozen autologous transfer cycles. We hypothesized that live birth and clinical pregnancy rates (CPR) would be higher in FET compared with fresh embryo transfers due to a combination of improved endometrial receptivity and improved embryo-endometrial synchrony in the FET cycles. Given that this is the largest such dataset ever published, we also hypothesized that the analysis would also provide important insights into factors that influence implantation rates in IVF and FET cycles.

## 2. Materials and Methods

We performed a cross-sectional analysis using data obtained from the MIVF patient database. Transfer of cleavage-stage embryos (day 2 embryos) was a standard practice at the MIVF at the time of the data collection. We, and the majority of IVF centers, now transfer and freeze blastocysts; however, given the unique size of the dataset, we anticipated that we would gain new insights into factors influencing embryo implantation.

According to the MIVF laboratory protocol, each embryo was evaluated twice before transfer. The first evaluation was performed 23-24 hr post-insemination/ICSI; referred to henceforth as the syngamy check. During this evaluation, embryos were assessed for the presence and number of cells (early cleavage (EC), nuclear envelope breakdown (NEBD), or 2 pronuclei (2PN)). The second evaluation was done on the morning of fresh embryo transfer (or before cryopreservation) on day 2 post-insemination/ICSI. Number of cells, degree of fragmentation, and multinucleation were assessed at the day 2 check. All embryos were cryopreserved using the slow freeze method (routine practice at the time of study) (22). The MIVF freeze-thaw protocols use post-thaw embryo culture to confirm resumed embryo development. Embryos for transfer in FET cycles are thawed the afternoon before the day of embryo transfer. These embryos are again evaluated twice; immediately after thawing for the number of surviving cells and a second time just prior to transfer in order to assess the resumption of mitosis and the total number of cells. Embryos are deemed suitable for transfer only if they survive the freeze-thaw process, defined as survival of ≥ 50% of the cells.

Information about embryo transfer cycles using cleavage-stage embryos was retrieved from the MIVF database. Given the large size of the database, we were able to enforce strict inclusion and exclusion criteria, but still retained a large dataset to analyze. A maximum of two stimulated cycles (cycle involving egg collection, embryo transfer of the best embryo, and freezing of remaining embryos for future use during thaw embryo cycles) were included in the analysis for each patient. Loss of a blastomere that is evident immediately post thaw is known to be associated with reduced embryo implantation potential (23). Therefore, we excluded those cycles using embryos where cell loss occurred during post-thaw embryo culture and evaluation. We also excluded cycles involving transfer of more than one embryo, use of donor gametes, or embryos with pre-implantation genetic testing. Women who had been pregnant from a previous IVF treatment were also excluded from the analysis.

We compared the clinical outcomes between fresh embryo transfer and FET cycles. The main outcome was LBR. A `live birth' is defined by the World Health Organization to be `the complete expulsion or extraction from its mother of a baby, irrespective of the duration of the pregnancy, which, after such separation, breathes or shows any other evidence of life'. The secondary outcome of this study was CPR. Clinical pregnancy is defined as the presence of fetal heart beat at first viability ultrasound (typically performed at gestational week 6 to 7 according to the MIVF protocol).

Stratification of cycles into fast- and slow-growing embryos was also performed. Slow cleavage-stage embryos were defined as those embryos that had 2PN during the syngamy check and were still at the 2-cell stage at the day 2 check. For the FET cycles, all embryos were at the 2-cells stage when frozen, and both cells survived the thawing process. Due to the post-thaw embryo culture protocol at MIVF for FET cycles, extra time allowed for development of frozen embryos, these embryos were half a day more advanced chronologically than their counterparts in fresh cycles. With the assumption that endometrial development is slightly advanced by COH, transferring frozen-thawed embryos that are half a day more advanced chronologically should improve embryo-endometrial developmental synchrony, leading to better CPR and LBR outcomes.

### Ethical consideration

This study was approved by the Royal Women's Hospital (RWH) Research Committee and RWH Human Research Ethics Committee (Project AQA19/15).

### Statistical analysis

Data were analyzed using the STATA 9.2 (StataCorp, Texas, USA) statistical and data analysis program. Continuous variables were examined in relation to relevant outcomes using the students *t* test, while binary variables were initially examined using the Chi-square test. Multivariate analysis was undertaken using logistic regression. For the purpose of model building, variables that reached p-value of < 0.1 were included in the final model. A P-value of < 0.05 was considered to be statistically significant in the output of the logistic regression analysis. Potential confounding factors including maternal age at egg collection, body mass index (BMI), cumulative embryo transfer cycle number (previous embryo transfer cycles included in the analysis), and embryo quality (embryo quality at the syngamy check, cell number,and embryo grade at day 2) were also examined.

## 3. Results

A total 10,744 cycles involving transfer of a single cleavage-stage embryo were identified between July 2009 and April 2015, comprising 7,014 fresh cycles and 3,730 FET cycles. Table I shows the main outcomes and potential confounders in the two groups. Surprisingly, both LBR and CPR were higher in the fresh cycle group compared to the FET group (LBR 19.13% vs 14.13%, p < 0.00; CPR 22.48% vs 16.25%, p < 0.00). Multiple pregnancy rates were low as only single embryo transfers were included in the study, and there was no statistical difference between the two groups. Compared to women in the fresh cycle group, women in the thaw cycle group were slightly younger (35.14 vs 35.51 years, p < 0.00), and more likely to have had an embryo transfer previously (2.68 vs 1.30, p < 0.00). There was no statistical difference for BMI (fresh vs frozen-thaw, 25.10 vs 24.98 kg/m2, p = 0.17) and fertilization methods (IVF 30.33 vs 31.05, ICSI 69.67 vs 68.95, p < 0.44) between the two groups. Embryo quality was better in the fresh cycle group, with more embryos being at the EC stage at the syngamy check (31.11 vs 19.65%, p < 0.00), higher cell number on day 2 (mean cell number 3.84 vs 3.63, p < 0.00), and better grade embryos (mean grade of embryos 1.87 vs 2.00, p <0.00). This reflects the practice of transferring the best embryo and freezing the remaining cohort of embryos during a stimulated cycle (Table I).

Multivariate analysis shows that women receiving a frozen-thawed embryo had a significantly lower LBR rate compared to those receiving a fresh embryo (OR 0.76, 95% CI 0.68-0.86, p < 0.00), after correcting for potential confounding factors - age, embryo quality (syngamy, cell number, embryo grade), fertilization method, cumulative ET and BMI (Table II). As expected, a higher pregnancy rate was observed in younger women (OR 0.91, 95% CI 0.90-0.93, p < 0.00) and those who had a better-quality embryo transferred (syngamy: OR 0.77, 95% CI 0.71-0.82, p < 0.00; cell number on day 2: OR 1.17, 95% CI 1.08-1.25, p < 0.00; embryo grade on day 2: OR 0.77, 95% CI 0.72-0.84, p < 0.00). Those who had embryo fertilized through ICSI had lower LBR compared to IVF (OR 0.89, 95% CI 0.80-1.0, p = 0.04). Previous cumulative embryo transfer number (OR 0.94, 95% CI 0.89-1.0, p = 0.06) and BMI of the women (OR 0.99, 95% CI 0.98-1.1, p = 0.2) did not have a statistically significant effect on LBR (Table II).

### Sub-analysis using slow-growing embryos

A total of 1,154 cycles involving transfer of a slow-growing cleavage-stage embryo were identified, of which 497 were fresh transfer cycles and 584 were FET cycles. Women in the fresh cycle group were on average 10 months older than those in the FET cycle group (37.29 vs 36.41 years, p < 0.00), and had had less embryo transfer cycles (1.39 vs 2.97 cycles, p < 0.00). There was no statistically significant difference in BMI, embryo fertilization method, or embryo grading between the two groups. Most importantly, there was no statistical difference between the fresh ET and the FET cycle groups in either LBR (6.40% vs 6.26%, p = 0.92) or CPR (8.10% vs 7.22%, p = 0.58) between the groups (Table III).

**Table 1:** Outcomes and comparison of potential confounders in the fresh ET and FET groups


	**Fresh cycles (n = 7014)**	**Frozen-thaw cycles (n = 3730)**	**P-value**
**Age (yr)**		35.51	35.14	< 0.0001
**Body mass index (kg/m ${}^{2}$)**		25.10	24.98	0.3474
**Cumulative ET (n = mean)**		1.30	2.68	< 0.0001
**Fertilization methods (%)**				
	**IVF**	30.33	31.05	0.44
	**ICSI**	69.67	68.95	0.44
**Syngamy check (%)**				
	**EC**	31.11	19.65	< 0.0001
	**NEBD**	39.67	33.46	< 0.0001
	**2PN**	29.22	46.89	< 0.0001
**Day 2 assessment**				
	**Number of cell on day 2 (mean)**	3.84	3.63	< 0.0001
	**Grade of embryos (mean)**	1.87	2.00	< 0.0001
**Grade of embryos (%)**				
	**Grade 1**	35.56	27.35	< 0.0001
	**Grade 2**	42.81	46.04	< 0.0001
	**Grade 3**	20.37	26.11	< 0.0001
	**Grade 4**	1.26	0.51	< 0.0001
**Life birth (%)**		19.13	14.13	< 0.0001
**Clinical pregnancy (%)**		22.48	16.25	< 0.0001
**Multiple pregnancy (%)**		1.12	2.09	0.107
Continuous variables were examined in relation to relevant outcomes using the students *t* test, while binary variables were initially examined using the Chi-square test. Cumulative ET (embryo transfer) refers to previous embryo transfer cycles included in the analysis. Syngamy check was the first evaluation performed 23-24 hr after insemination/ICSI when the embryos were assessed for the presence and number of cells (EC = early cleavage, NEBD = nuclear envelope breakdown, or 2PN = 2 pro-nuclei). Embryos were evaluated again on day 2 post insemination/ICSI. Number of cells, degree of fragmentation, and multinucleation (grading of embryos) were assessed at the day 2 check				

**Table 2:** Multivariate analysis showing the effect on LBR of fresh versus frozen cycle, age, embryo quality, fertilization method, and cumulative ET and BMI


	**Odds ratio**	**Standard error**	**z**	**P > |z|**	**[95% CI]**	
**Cycle type (fresh ET vs FET)**	0.7625669	0.0447572	-4.62	0.000	0.6797021	0.8555341
**Age**	0.9144266	0.0054659	-14.97	0.000	0.9037761	0.9252027
**Syngamy (EC, NEBD or 2PN)**	0.7656162	0.0281109	-7.27	0.000	0.7124557	0.8227434
**Cell number on day 2**	1.1618480	0.0441235	3.95	0.000	1.0785080	1.2516290
**Embryo grade on day 2**	0.7765594	0.0283183	-6.93	0.000	0.7229937	0.8340938
**Fertilization method (IVF vs ICSI)**	0.8910954	0.0505859	-2.03	0.042	0.7972655	0.9959681
**Cumulative ET**	0.9443254	0.0288636	-1.87	0.061	0.8894150	1.0026260
**BMI**	0.9926360	0.0057259	-1.28	0.200	0.9814767	1.0039220
Multivariate analysis was undertaken using logistic regression. Syngamy check was the first evaluation performed 23-24 hr after insemination/ICSI when the embryos were assessed for the presence and number of cells (EC = early cleavage, NEBD = nuclear envelope breakdown, or 2PN = 2 pro-nuclei). Embryos were evaluated again on day 2 post insemination/ICSI. Number of cells, degree of fragmentation, and multinucleation (grading of embryos) were assessed at the day 2 check. Cumulative ET (embryo transfer) refers to previous embryo transfer cycles included in the analysis						

**Table 3:** Comparison of fresh ET and FET cycles using slow-growing embryos


**Characteristic**	**Fresh cycles (n = 531)**	**Thaw cycles (n = 623)**	**P-value**
**Maternal age (years)**	37.29	36.41	0.0016
**Body Mass Index (kg/m${}^{2}$)**	25.32	25.11	0.5264
**ICSI (%)**	70.62	67.42	0.241
**Grade of embryo (mean)**	1.89	1.89	0.984
**Grade of embryo**			
**Grade 1 (%)**	34.65	33.87	0.968
**Grade 2 (%)**	42.75	44.14	0.968
**Grade 3 and above (%)**	22.03	21.51	0.968
**Cumulative ET**	1.39	2.97	< 0.0001
**Clinical pregnancy (%)**	8.10	7.22	0.577
**Life birth (%)**	6.40	6.26	0.921
Slow cleavage-stage embryos were defined as those embryos that had 2 pronuclei during the syngamy check at 23-24 hr and were still at the 2-cell stage at the day 2 check. For FET cycles, all embryos were at the 2-cell stage when frozen, and both cells survived the thawing process. Cumulative ET (embryo transfer) refers to previous embryo transfer cycles included in the analysis. Grade of embryos is assessed on day 2 post-insemination/ICSI for the degree of fragmentation and multinucleation. Continuous variables were examined in relation to relevant outcomes using the students *t* test, while binary variables were initially examined using the Chi-square test			

## 4. Discussion

This cross-sectional analysis of outcomes from fresh vs. FETs is the first to only include single embryo transfer cycles; and with 7,014 fresh cycles and 3,730 FET cycles, it is also the largest study of this type to be published. The primary finding from our analysis was a significantly lower LBR and CPR in FET cycles compared to fresh cycles, using single autologous cleavage-stage embryos. Multivariate analysis identified six variables that impacted significantly on LBR (Table II). Controlling for these confounding factors confirmed the finding of lower LBR in FET cycles. These results do not support our hypothesis that LBR and CPR would be higher in FET compared with fresh embryo transfers due to a combination of improved endometrial receptivity and improved embryo-endometrial synchrony in the FET cycles. Rather, they suggest that any potential gains in LBR due to improved endometrial receptivity and improved embryo-endometrial synchrony are lost with FET, presumably due to embryo damage caused during the freeze-thaw process.

A secondary finding from this study was a sub-analysis of cycles with slow-growing embryos. We hypothesized that embryo-endometrial developmental synchrony would be improved in FET compared to fresh cycles using slow embryos due to the post-thaw embryo culture protocol. In our analysis of 1,154 cycles using slow embryos, there was no statistical difference in LBR or CPR between the two groups. We interpret this result as showing that the reduction in LBR due to freeze-thawing damage to the embryo seen in fast-growing embryos is fully compensated for by the improved synchrony in the slow-growing embryos. Slower-growing embryos typically result in lower PR in fresh cycles, potentially due to reduced embryo viability and increased embryo-endometrial asynchrony. Taken together, the results from this study confirm the positive influence of younger maternal age, better embryo quality, faster embryo development and improved embryo-endometrial developmental synchrony on LBR, while also demonstrating that embryo freezing has a negative impact on LBR.

There have been a limited number of published studies involving smaller sample sizes comparing clinical outcomes between fresh and FET cycles. While each of these provides some insight into the relative contributions of embryo viability, uterine receptivity, and embryo-endometrial synchrony to LBR, the individual studies are not all directly comparable and their findings not in complete agreement.

The study published by Shapiro and colleagues mentioned earlier in this paper, found increased CPR in the FET cycle cohorts. It is important to note that the best blastocysts of the cohort were transferred in fresh cycles, while only good-quality embryos (expanded supernumerary blastocysts) were selected for cryopreservation and subsequent use in FET cycles. Embryo selection was a potential confounder in this study, which was acknowledged by the authors in their article (20). Two subsequent prospective randomized studies by the same group comparing fresh blastocyst transfer and oocyte cryopreservation (with subsequent blastocyst transfer grown from the frozen-thawed oocytes) has shown a significantly greater CPR in the oocyte cryopreservation group in normal responders, but no statistical difference in high responders (24, 25). Overall, these studies support the hypothesis that FET improves CPR, presumably through improving uterine receptivity and/or embryo-endometrial synchrony. There is no evidence from these studies for a negative effect from freeze-thawing on embryo viability, possibly due to factors such as reduced blastomere size in the blastocyst compared to a cleavage-stage embryo. An alternative explanation is that by selecting embryos that have reached the expanded blastocyst stage, it is possible to eliminate all the nonviable embryos that appeared “normal” at the cleavage stage.

In two prospective cohort studies involving use of cleavage-stage embryos and large number of cycles, LBR was lower in FET cycles when compared to fresh cycles (26, 27). Four other randomized controlled trials using cleavage-stage embryos have also found no difference in LBR between fresh and FET cycles (28-31). The first trial involved 2,157 young (20-35 year old) women undergoing IVF/ICSI treatment due to tubal and/or male factors infertility (28). In the second trial, 782 infertile women were randomly assigned to fresh transfer or frozen embryos on day 3. In the FET group, only good-quality embryos (grade 1 or 2) were used (29). The last two studies involved women at risk of OHSS (30, 31). All studies involved the transfer of multiple embryos (28-31). Differences between the above studies and our study include patient population, number of embryos transferred, and embryo freezing method. Any or all of these factors could have contributed to the different clinical outcomes between these studies (no difference in LBR between fresh and FET cycles) and ours (higher LBR in fresh cycles).

While studies involving blastocyst transfer have suggested a better LBR with FET cycles (20, 24), our results and other studies using cleavage-stage embryos have not found the same (28-30). A major difference between protocols is that cleavage-stage embryos are cryopreserved according to their chronological age (day 2), while blastocysts are only cryopreserved if they reached the desired developmental stage of expanded blastocyst. The developmental stage of embryos is an important factor when considering embryo-endometrial synchrony. The cleavage-stage embryo freezing protocol has not been designed to correct for any potential asynchrony between developmental stage for the embryo and endometrium. There may be clinical benefit in the future to considering developmental stage rather than chronological age when thawing cleavage-stage embryos for transfer.

In conclusion, in this retrospective study of 10,744 IVF cycles, we were not able to support our hypothesis that LBR and CPR would be higher in FET compared with fresh embryo transfers. Multivariate analysis with correction for five significant variables showed a significantly higher LBR rate in cycles using fresh embryos compared to frozen-thawed embryos. In our sub-analysis of cycles involving slow-growing embryos, we interpreted the fact that there was no statistical difference in LBR between the fresh and FET groups as evidence for improved embryo-endometrial synchrony in the FET cycles. The large size of our dataset coupled with the strict inclusion and exclusion criteria have allowed us to identify six variables that significantly impact LBR, in addition to the results from our sub-analysis on slow-growing embryos which provides data to support the view that embryo-endometrial developmental synchrony is important. While most IVF units have moved to blastocysts transfer and freezing, the findings from this study on two-day old embryos remain highly relevant for understanding and optimizing LBR in today's IVF clinic.

##  Conflict of Interests

None declared.
